# Research on the Protective Effects and Mechanisms of Gallic Acid Against Cognitive Impairment Induced by Chronic Sleep Deprivation

**DOI:** 10.3390/nu17203204

**Published:** 2025-10-12

**Authors:** Xiangfei Zhang, Jingwen Cui, Jing Sun, Fengzhong Wang, Bei Fan, Cong Lu

**Affiliations:** 1 Institute of Food Science and Technology, Chinese Academy of Agricultural Sciences, Key Laboratory of Agro-Products Processing, Ministry of Agriculture and Rural Affairs, Beijing 100193, China; 82101235017@caas.cn (X.Z.); 18734021485@163.com (J.C.); ycsunjing2008@126.com (J.S.); wangfengzhong@sina.com (F.W.); 2 Institute of Food and Nutrition Development, Ministry of Agriculture and Rural Affairs, Beijing 100081, China

**Keywords:** gallic acid, dietary polyphenol, chronic sleep deprivation, cognitive impairment, oxidative stress, neuroinflammation

## Abstract

**Background**: Gallic acid (GA) is a dietary polyphenol widely found in walnuts, tea leaves, and grapes, and it is recognized for its potent antioxidant and anti-inflammatory properties. Chronic sleep deprivation (CSD) is known to disrupt redox balance, promote neuroinflammation, and impair cognition, while effective nutritional strategies to mitigate these effects remain scarce. This study was designed to evaluate the protective potential of GA against CSD-induced cognitive deficits in mice and to elucidate the underlying mechanisms. **Methods**: Seventy-two male ICR mice were randomly allocated to six groups, including control, CSD model, Ginkgo biloba extract, and GA at three doses (50, 100, and 200 mg/kg). After 28 days of treatment, cognitive performance was assessed using the open field test (OFT), novel object recognition (NOR), step-through passive avoidance (ST), and Morris water maze (MWM). Redox status and inflammatory mediators were determined by ELISA, while the hippocampal expression of proteins related to antioxidant defense and NF-κB signaling was analyzed by Western blotting. **Results**: GA supplementation improved exploratory activity, recognition memory, and spatial learning in the CSD mice. Biochemical evaluation revealed that total antioxidant capacity (T-AOC) and superoxide dismutase (SOD) activity were restored, while malondialdehyde (MDA) levels, an indicator of lipid peroxidation, were reduced. These changes were accompanied by decreased circulating concentrations of interleukin-1β (IL-1β), interleukin-6 (IL-6), and tumor necrosis factor-α (TNF-α). At the molecular level, GA enhanced the expression of Nrf2, HO-1, and NQO1, while inhibiting p-p65, iNOS, and COX2 in the hippocampus. **Conclusions**: These findings demonstrate that GA alleviates CSD-induced cognitive deficits through the activation of the Nrf2/HO-1 antioxidant pathway and inhibition of NF-κB–mediated inflammatory responses. Thus, GA may represent a promising nutraceutical candidate for maintaining cognitive health under chronic sleep loss.

## 1. Introduction

Cognitive impairment, manifested as deficits in learning, memory, attention, and executive function [1], has emerged as a growing global health concern and frequently precedes the onset of neurodegenerative diseases, such as Alzheimer’s and Parkinson’s disease [2,3]. Among lifestyle-related risk factors, chronic sleep deprivation (CSD)—arising from irregular work schedules, prolonged stress, and excessive night-time screen exposure—has been identified as a major contributor to cognitive decline [4,5]. CSD not only disrupts sleep quality, but it also provokes oxidative stress, neuroinflammation, and synaptic dysfunction, thereby accelerating aging processes and increasing vulnerability to mood disorders and neurodegenerative conditions [6,7]. Given that pharmacological treatments provide only short-term symptomatic relief and are frequently limited by side effects, nutritional strategies are increasingly recognized as safer and more sustainable approaches to counteract CSD-induced cognitive dysfunction [8,9].

Polyphenols are well-established dietary bioactive compounds that exert a wide range of health-promoting effects in humans [10,11]. Gallic acid (GA), a representative polyphenol abundant in walnuts, tea leaves, grapes, and pomegranates [12], has attracted considerable attention for its diverse biological activities. GA possesses antioxidant [13], anti-inflammatory [14], antimicrobial [15], and anticancer properties, among which its antioxidant effect is particularly prominent. This antioxidant capacity underpins most of its documented health benefits by neutralizing reactive oxygen species (ROS) and enhancing endogenous defense mechanisms.

Although extensive research has elucidated the antioxidant mechanisms, pharmacological potential, and food-related applications of GA, its impact on sleep deprivation-induced cognitive dysfunction remains largely unexplored. Our preliminary studies demonstrate that GA attenuates oxidative stress-induced damage in SH-SY5Y neuroblastoma cells, highlighting its potential neuroprotective effects at the cellular level. However, whether these in vitro findings translate to in vivo protection against cognitive impairments caused by sleep loss remains uncertain.

Therefore, this study employed a CSD mouse model to assess GA supplementation’s impact on cognition and explored its molecular mechanisms. By combining behavioral assessments with biochemical analyses, this study offers fresh perspectives on the functional value of GA, and it also offers a theoretical basis for its further development as a dietary polyphenol with potential applications in cognitive health management and functional foods.

## 2. Materials and Methods

### 2.1. Materials

Gallic acid (GA) was obtained from Shanghai Yuanye Bio-Technology Co., Ltd. (Shanghai, China). Ginkgo biloba extract (approval no. HJ20140768) was provided by Dr. Willmar Schwabe GmbH & Co. KG (Karlsruhe, Germany); each tablet contained 40 mg of extract, which were standardized to 9.6 mg flavonoid glycosides and 2.4 mg terpene lactones. Biochemical kits for T-AOC, SOD, MDA, TNF-α, IL-6, and IL-1β were purchased from Nanjing Jiancheng Bioengineering Institute (Nanjing, China). The antibodies for Nrf2, HO-1, NQO1, p-p65, p65, iNOS, COX2, and β-actin were obtained from Proteintech (Chicago, IL, USA); secondary antibodies were from Abcam (Cambridge, UK).

### 2.2. Grouping and Management of Animals for the CSD Model

Seventy-two male Institute of Cancer Research (ICR) mice (18–22 g) of specific pathogen-free (SPF) status were supplied by the Hunan Prima Pharmaceutical Research Center Co., Ltd. (Changsha, China) (production license number: SCXK [Xiang] 2021-0002; use license numbers: SYXK [Xiang] 2020-0015 and SYXK [Xiang] 2025-008; certificate numbers: 430727251101136628 and 430727251101315256). This study strictly adhered to the ethical guidelines of the 3Rs (Replacement, Reduction, and Refinement) framework, as well as the globally recognized protocols for the humane treatment of research animals. The Institutional Animal Care and Use Committee at Hunan Prima Pharmaceutical Research Center Co., Ltd. thoroughly reviewed and approved all experimental protocols, thereby ensuring compliance with international standards for the ethical treatment of animals. All procedures were performed in accordance with internationally recognized animal welfare guidelines. The mice were housed in a controlled environment maintained at 20–24 °C, approximately 55% relative humidity (±10%), with a consistent 12 h light/dark cycle. The animals were kept in cages containing six mice per cage, with ad libitum access to food and water. Following a three-day acclimation period, the mice were randomly assigned to six experimental groups (12 animals each) based on body weight:

1. CON (no stress, daily oral vehicle administration);
2. CSD (exposed to the CSD regimen and receiving oral vehicle administration daily);
3. CSD + GBE (GBE orally given to the CSD mice at 40 mg/kg/day, positive control);
4. CSD + GA-L (CSD mice orally administered GA, 50 mg/kg/day);
5. CSD + GA-M (CSD mice orally administered GA, 100 mg/kg/day);
6. CSD + GA-H (CSD mice orally administered GA, 200 mg/kg/day).

Gallic acid (GA) was administered orally at doses of 50, 100, or 200 mg/kg. These dose levels were selected based on previous reports that showed that GA, within the range of 50–200 mg/kg, is effective and well tolerated in rodent models of oxidative stress and cognitive dysfunction [16,17,18].

Ginkgo biloba extract (GBE, EGb 761^®^) was included as a positive control based on the clinical consensus supporting its efficacy in mild cognitive impairment [19]. For preclinical use, a dose of 40 mg/kg/day has been reported to enhance recognition memory and hippocampal neurogenesis in mice [20]. This regimen has also been applied in our laboratory under comparable conditions, confirming its suitability as a benchmark treatment [21].

All doses of GA (50–200 mg/kg/day) and GBE (40 mg/kg/day) were well tolerated under the present conditions, with no signs of abnormal behavior, diarrhea, weight loss, or mortality observed throughout the experimental period.

### 2.3. Induction of the CSD Mouse Model

The CSD model was developed using a rolling sleep interruption device. Upon procurement, the rodents were randomly divided into groups and acclimatized for a 3-day period. Preventive treatment was administered for 14 days prior to modeling. The experiments were conducted in a soundproof and controlled animal facility. Food and water bottles were placed inside the apparatus to ensure free access during the procedure. Except for the control group, all subjects underwent placement within the device, which was set to operate under these specific settings: a rotation speed of 1 r/min and a single rotation interspersed with a 2 min rest, with directional shifts at random intervals.

**Figure 1:**
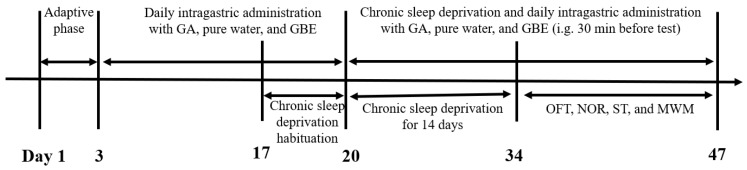
The experimental schedule.

From Days 17–20, the animals underwent a 3 h daily deprivation protocol at a consistent time for acclimation. Following this period, from Day 21 to Day 34, the same parameters were applied continuously for 14 days to induce CSD. GA, pure water, and GBE administration were maintained throughout both the adaptation and modeling periods. Figure 1 depicts the experimental design.

### 2.4. Behavioral Tests

#### 2.4.1. Open Field Test (OFT)

On Day 35, movement was evaluated via the OFT [22]. Approximately 30 min after treatment administration, the mice were placed in the center of an open-field box (40 × 40 × 35 cm) to assess spontaneous locomotor activity. After a 3 min adaptation period, the system automatically recorded their movements during a 5 min test session. Indicators of general activity, including total distance traveled, mean velocity, and the center-to-periphery time ratio, were extracted to evaluate spontaneous exploration and to determine whether the treatment influenced locomotor performance.

#### 2.4.2. Novel Object Recognition Test (NOR)

The NOR test was used to assess short-term recognition memory [23]. The test consisted of three phases: habituation, familiarization, and test. During the habituation phase (Days 36–38), the mice were placed in the empty arena (40 × 50 × 50 cm) for 10 min daily. On Day 39, the familiarization phase was performed by placing two identical objects in fixed positions, and the mice were allowed to explore for 5 min. After a 30 min retention interval, one object was replaced with a novel object of similar size but different shape and material, and the mice were allowed another 5 min exploration. During the test, exploratory behavior was videotaped and the exploration time was recorded. Recognition memory performance was evaluated by the discrimination index (DI = [T_N_ − T_F_]/[T_N_ + T_F_]), where T_N_ is the time spent exploring the novel object, and T_F_ the time spent exploring the familiar object.

#### 2.4.3. Step-Through Test (ST)

The ST test was conducted on Days 40 and 41 to evaluate learning and memory performance [24]. On Day 40 (acquisition phase), the mice were tested 30 min after treatment. Each mouse was placed in the illuminated compartment of the apparatus with its back to the entrance. After a 3 min adaptation, entry into the dark chamber triggered a 0.5 mA foot shock for 5 s, followed by removal after a 5 min session. On Day 41 (retention phase), the same procedure was repeated 30 min after treatment, and the mice received the shock immediately upon entering the dark chamber. Latency to first entry and error frequency were recorded as indices of memory retention.

#### 2.4.4. Morris Water Maze Test (MWM)

From Days 42 to 47, spatial cognition was evaluated using the MWM [25,26]. The apparatus consisted of a black circular tank filled with water made opaque with non-toxic ink and maintained at 22 ± 2 °C. A dark cylindrical platform (6 cm diameter) was placed 1 cm below the surface in the target quadrant. A camera mounted above the pool was connected to a computerized tracking system to record the swimming distance, speed, and latency.

The task included a 5-day acquisition phase followed by a probe trial on Day 6. During acquisition, the mice received three trials per day, each lasting a maximum of 90 s, to locate the hidden platform. Escape latency was recorded as the primary measure of learning. Mice that failed to find the platform within 90 s were guided to it and allowed to remain for 10 s. On the probe trial, the platform was removed, and each mouse was released from the quadrant opposite the previous platform location. The number of crossings over the former platform site within 90 s was measured as the index of memory retention.

### 2.5. Sample Collection

On the day following the final behavioral test, the mice were anesthetized with 10% chloral hydrate and euthanized by decapitation in accordance with institutional animal care guidelines. Blood was collected immediately, allowed to clot at 4 °C, and centrifuged to obtain serum. The brains were then rapidly removed, and the hippocampi were dissected on ice. Both serum and hippocampal samples were stored at −80 °C for subsequent analysis.

### 2.6. Biochemical and Immunoassays

Commercial kits were applied to evaluate the serum and hippocampal tissue T-AOC, SOD activity, and MDA content. The levels of IL-1β, IL-6, and TNF-α in serum and in the hippocampal tissue were analyzed using ELISA.

### 2.7. Western Blotting Analysis

Hippocampal proteins were extracted using RIPA buffer containing protease and phosphatase inhibitors. Protein concentrations were measured with the BCA assay, and equal amounts of protein were resolved by SDS-PAGE under denaturing conditions and then transferred to PVDF membranes. After blocking, the membranes were incubated overnight at 4 °C with primary antibodies against Nrf2, HO-1, NQO1, phosphorylated p65, p65, iNOS, COX2, and β-actin, and this was followed by incubation with HRP-conjugated secondary antibodies. Protein bands were visualized using enhanced chemiluminescence (ECL) reagents and captured with a digital imaging system. Band intensities were quantified using Image-Pro Plus 6.0 (Media Cybernetics, Rockville, MD, USA), with β-actin serving as an internal loading control to normalize protein expression levels.

### 2.8. Data Analysis

SPSS 24.0 (SPSS Inc., Chicago, IL, USA) was used for statistical analysis. Data are given as the mean ± SEM. Repeated-measures, two-way ANOVA was applied for MWM latency, and one-way ANOVA with LSD post hoc tests was used for the remaining variables. Statistical significance was set at *p* < 0.05.

## 3. Results

### 3.1. Effects of GA on Body Weight in Mice

**Figure 2:**
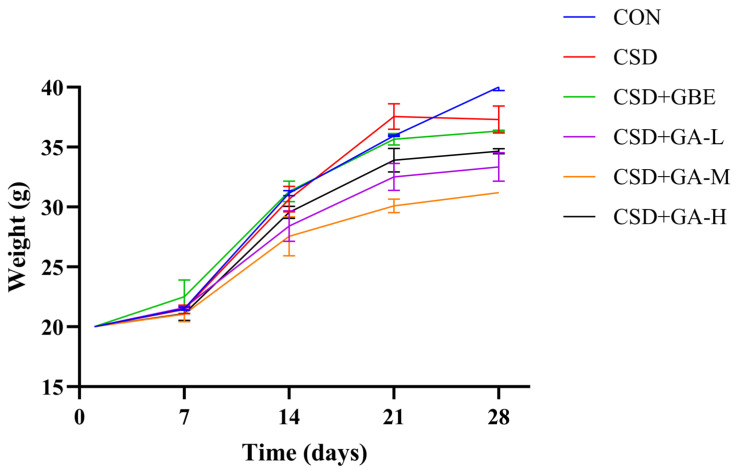
Body weight changes of the CSD mice during the experimental period under different treatments (mean ± SEM, *n* = 12).

Body weight increased progressively in all groups during the 28-day observation period (Figure 2). In the pre-intervention phase (Days 0–14), no significant differences were observed among the groups. After the onset of CSD modeling (Days 14–28), the CSD group showed a markedly slower rate of weight gain relative to the control group. The CSD+GBE group maintained a trajectory close to that of the control group, indicating that GBE attenuated the stress-induced suppression of body weight gain and helped preserve a stable growth curve under chronic stress conditions. In the GA-treated groups, the slopes of body weight gain were broadly similar during this period; however, the overall changes did not follow a strictly linear dose–response. Specifically, the GA-M group showed lower mean body weight than GA-L, whereas GA-H partially recovered toward the GA-L level. This non-linear pattern suggests that GA administration did not exert a monotonic effect on body weight and that its more consistent effects were reflected in the behavioral and biochemical outcomes rather than in the growth parameters.

### 3.2. Effects of GA Intervention on Locomotor and Exploratory Behaviors in CSD Mice Assessed by the OFT

**Figure 3:**
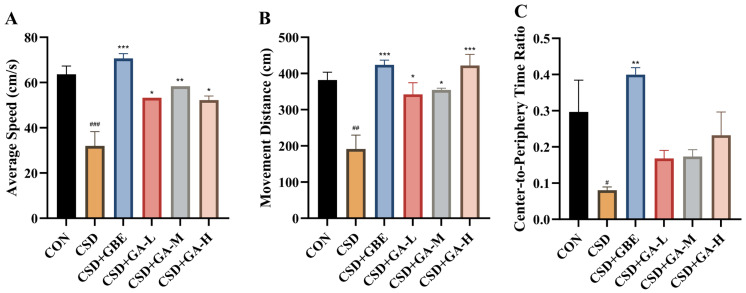
Locomotion and exploration in the CSD mice during the OFT after GA administration. (**A**) Average speed, (**B**) movement distance, and (**C**) center-to-periphery time ratio. Results are shown as the mean ± SEM (*n* = 12). ^#^ *p* < 0.05, ^##^ *p* < 0.01, and ^###^ *p* < 0.001 vs. CON; * *p* < 0.05, ** *p* < 0.01, and *** *p* < 0.001, vs. CSD.

In the OFT, the CSD mice exhibited markedly reduced locomotor activity compared with the controls, as indicated by decreased average speed (Figure 3A) and movement distance (Figure 3B). Moreover, the center-to-periphery time ratio was substantially lower in the CSD group (Figure 3C), suggesting reduced exploratory behavior under CSD. GBE intervention significantly improved these parameters, restoring them toward control levels. GA intervention also showed dose-related effects: the low- and medium-dose groups partially increased locomotor activity versus the CSD group, while the high-dose group demonstrated more evident improvements in both movement and central exploration.

### 3.3. Effects of GA on Object Recognition in CSD Mice

**Figure 4:**
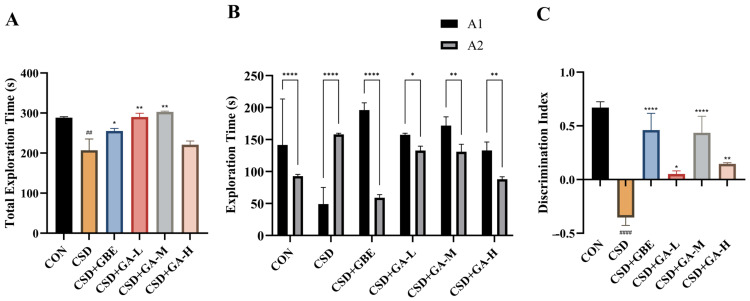
Effects of GA on exploration behavior and recognition memory in the NOR test. (**A**) Total exploration time of objects during the test phase. (**B**) Exploration time spent exploring the novel object (A1) and the familiar object (A2) across groups. (**C**) Discrimination index (DI). Results are shown as the mean ± SEM (*n* = 12). ^##^ *p* < 0.01 and ^####^ *p* < 0.0001 vs. CON; * *p* < 0.05, ** *p* < 0.01, and **** *p* < 0.0001 vs. CSD. In Panel B, the same star symbols placed above the connecting brackets denote the within-group comparisons between A1 and A2.

In the NOR test, the CSD mice exhibited a marked reduction in total exploration time compared with the CON group (Figure 4A, ^##^ *p* < 0.01), indicating impaired exploratory activity. Treatment with GA (50–100 mg/kg) or GBE significantly increased the total exploration time relative to the CSD mice (* *p* < 0.05, ** *p* < 0.01), suggesting a partial restoration of exploratory drive.

When comparing exploration of novel (A1) and familiar (A2) objects, the CON mice displayed a significant preference for the novel object (**** *p* < 0.0001, Figure 4B). This preference was abolished in the CSD group, as no significant difference was observed between A1 and A2, indicating recognition memory deficits. In contrast, the GBE- and GA-treated mice (all doses) showed a restored preference for the novel object, with significantly greater exploration of A1 than A2 (* *p* < 0.05, ** *p* < 0.01, and **** *p* < 0.0001).

Consistently, the discrimination index (DI) was significantly decreased in the CSD group compared with the CON mice (Figure 4C, **** *p* < 0.0001), whereas GBE and GA administration markedly improved DI values compared with the CSD controls (* *p* < 0.05, ** *p* < 0.01, and **** *p* < 0.0001). These results demonstrate that GA effectively ameliorates CSD-induced recognition memory impairment, comparable to the effects of GBE.

### 3.4. Effects of GA Intervention on Learning and Memory Performance in CSD Mice Assessed by the ST

**Figure 5:**
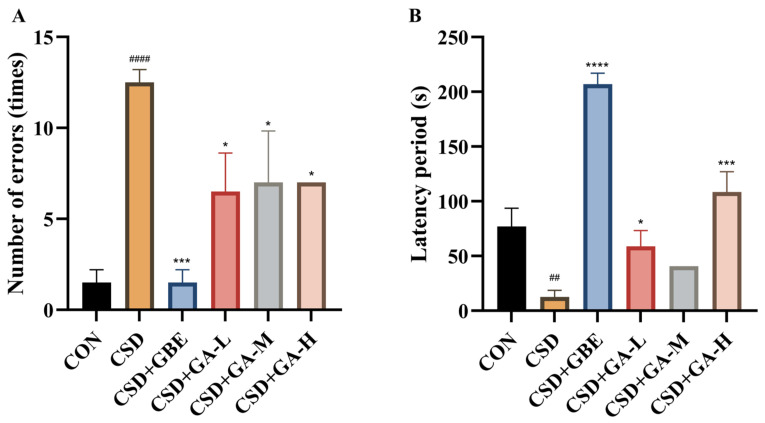
Learning and memory performance in the CSD mice assessed by the ST test after GA administration. (**A**) Number of errors. (**B**) Latency period. Results are shown as the mean ± SEM (*n* = 12). ^##^ *p* < 0.01 and ^####^ *p* < 0.0001 vs. CON; * *p* < 0.05, *** *p* < 0.001 and **** *p* < 0.0001 vs. CSD.

In the ST test, compared with the controls, the CSD mice displayed pronounced deficits in learning and memory. Specifically, the number of errors was markedly increased (Figure 5A) and the latency period was significantly shortened (Figure 5B). GBE intervention effectively reduced error frequency and prolonged latency, restoring performance close to control levels. GA intervention also alleviated cognitive deficits: all GA-treated groups showed fewer errors relative to the CSD group, with the high-dose group displaying more prominent improvements in latency extension. These findings indicate that GA supplementation mitigated CSD-induced impairments in associative learning and memory.

### 3.5. Effects of GA on Spatial Learning and Memory in CSD Mice Assessed by the MWM Test

In the MWM training phase (Table 1), the control mice showed a progressive decline in escape latency during training, whereas the CSD mice maintained prolonged latencies throughout the acquisition phase and failed to improve, indicating impaired spatial learning. GBE treatment effectively shortened latencies, reaching values close to controls by the final day. Gallic acid also improved learning, with the medium dose producing the most evident reduction, the high dose showing moderate benefit, and the low dose exerting only limited effects. In the subsequent probe trial (Table 2), the control mice spent significantly more time in the target quadrant, whereas the CSD mice exhibited no target preference, reflecting impaired memory retention. GBE restored target quadrant preference, and gallic acid treatment also increased time in the target quadrant, with the medium dose showing the most robust recovery, the high dose a partial improvement, and the low dose only minor changes.

**Figure 6:**
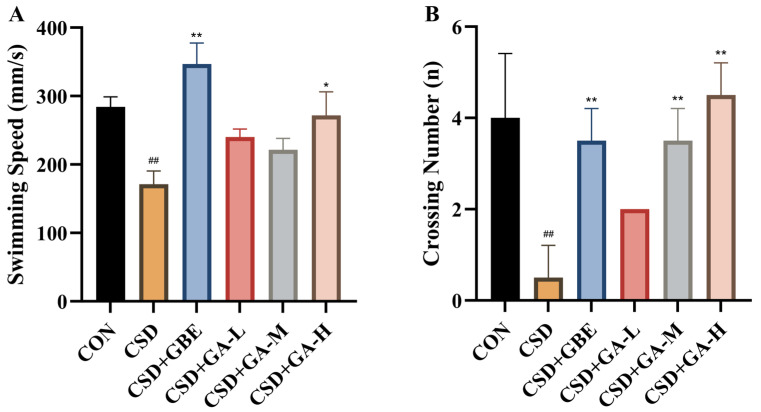
Spatial learning and memory of the CSD mice measured in the MWM probe test after GA administration. (**A**) Swimming speed. (**B**) Crossing number. Results are shown as the mean ± SEM (*n* = 12). ^##^ *p* < 0.01 vs. CON; * *p* < 0.05, ** *p* < 0.01 vs. CSD.

In the probe test (Figure 6), the CSD mice showed a marked reduction in swimming speed and platform crossing number compared with the control group, indicating impaired motivation and spatial memory retrieval. GBE treatment significantly increased both parameters, restoring them to levels comparable with controls. Gallic acid also improved performance: the low and high doses increased swimming speed relative to the CSD mice, while the medium dose showed only a slight, non-significant change. For platform crossings, both the medium and high doses significantly enhanced the number of target platform crossings compared with CSD, with the high dose showing the strongest effect, whereas the low dose produced a moderate improvement.

### 3.6. Effects of GA on Oxidative Stress Markers in Serum and Hippocampus of CSD Mice

**Figure 7:**
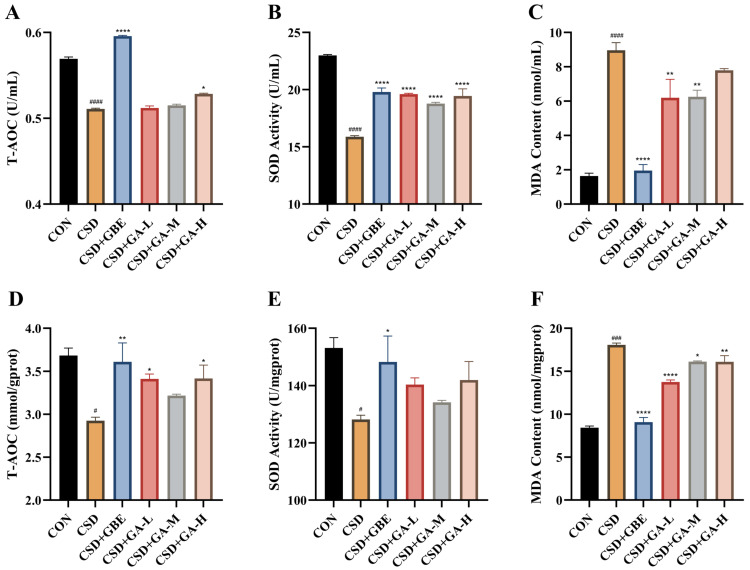
Effects of GA on oxidative stress indices in serum and in the hippocampus of the CSD mice. (**A**–**C**) Serum T-AOC, SOD activity, and MDA content. (**D**–**F**) Hippocampal T-AOC, SOD activity, and MDA content. Results are shown as the mean ± SEM (*n* = 12). ^#^ *p* < 0.05, ^###^ *p* < 0.001 and ^####^ *p* < 0.0001 vs. CON; * *p* < 0.05, ** *p* < 0.01, and **** *p* < 0.0001 vs. CSD.

Oxidative stress was evaluated by measuring the T-AOC, SOD, and MDA levels in serum and in the hippocampal tissues (Figure 7A–F). The CSD mice exhibited a pronounced oxidative disturbance, characterized by reduced antioxidant capacity and elevated lipid peroxidation compared with the control group. Specifically, both serum and hippocampal T-AOC and SOD activity were significantly decreased, while MDA levels were markedly increased. GBE administration effectively improved these alterations, restoring antioxidant indices and lowering MDA accumulation. GA treatment also mitigated CSD-induced oxidative stress. All GA groups showed significant improvements compared with the CSD group. The medium-dose GA group produced the most consistent recovery of T-AOC and SOD activity, whereas both medium- and high-dose groups reduced MDA levels more prominently, indicating that higher GA exposure exerts stronger effects than the low dose.

### 3.7. Effects of GA on the Nrf2/HO-1 Signaling Pathway in the Hippocampus of CSD Mice

**Figure 8:**
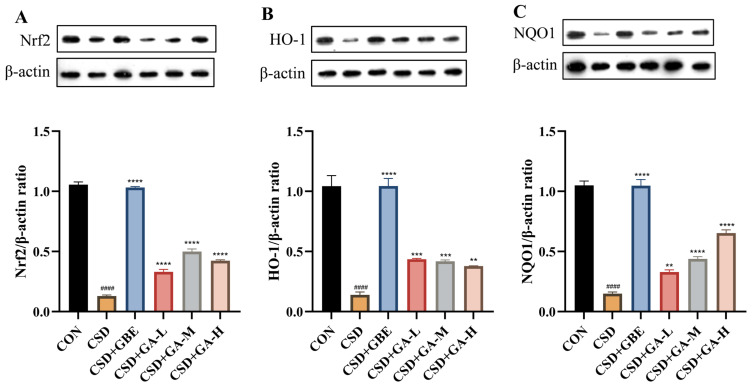
Serum cytokine concentrations in the CSD mice after GA administration. (**A**) Nrf2/β-actin; (**B**) HO-1/β-actin; (**C**) NQO1/β-actin. Results are shown as the mean ± SEM (*n* = 3). ^####^ *p* < 0.0001 vs. CON; ** *p* < 0.01, *** *p* < 0.001 and **** *p* < 0.0001 vs. CSD.

To further clarify the molecular basis of GA’s antioxidant actions, we assessed the Nrf2, HO-1, and NQO1 levels in the hippocampus (Figure 8). Compared with the controls, the CSD mice exhibited markedly decreased protein expression levels of Nrf2, HO-1, and NQO1 (Figure 8A–C), indicating suppression of the endogenous antioxidant defense system. GBE treatment significantly upregulated these proteins, restoring them toward control levels. GA supplementation also increased the Nrf2, HO-1, and NQO1 expression compared to the CSD group, with the medium and high doses showing particularly strong effects. These findings indicate that GA helps counteract oxidative stress, in part, through the Nrf2/HO-1 signaling pathway activation.

### 3.8. Effects of GA on Pro-Inflammatory Cytokine Levels in Serum and Hippocampus of CSD Mice

**Figure 9:**
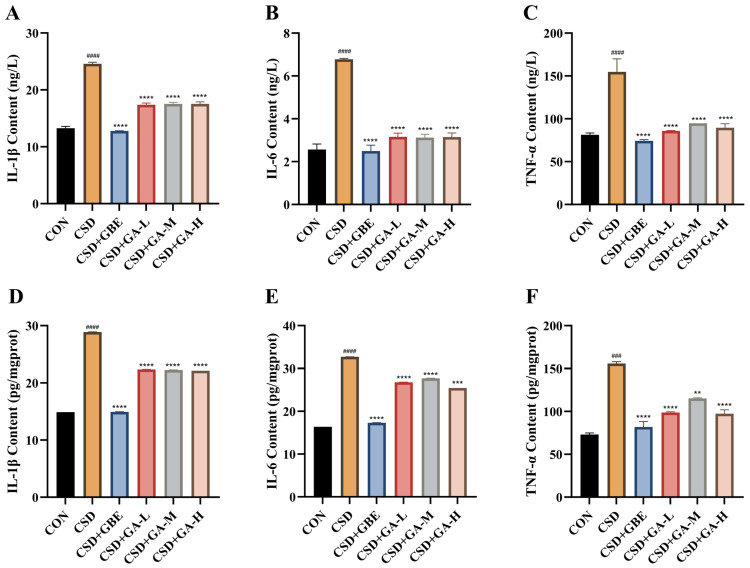
Effects of GA on inflammatory cytokine levels in serum and in the hippocampus of the CSD mice. (**A**–**C**) Serum IL-1β, IL-6, and TNF-α concentrations. (**D**–**F**) Hippocampal IL-1β, IL-6, and TNF-α concentrations. Results are shown as the mean ± SEM (*n* = 12). ^###^ *p* < 0.001 and ^####^ *p* < 0.0001 vs. CON; ** *p* < 0.01, *** *p* < 0.001 and **** *p* < 0.0001 vs. CSD.

To evaluate inflammatory responses, the IL-1β, IL-6, and TNF-α levels were measured in serum and in the hippocampal tissues (Figure 9A–F). Compared with the control group, the CSD mice exhibited markedly increased concentrations of all three cytokines in both the serum (Figure 9A–C) and hippocampus (Figure 9D–F), indicating robust systemic and central inflammation induced by sleep deprivation. GBE treatment significantly suppressed cytokine production, restoring levels close to those observed in the controls. GA supplementation also reduced inflammatory activity: the mice receiving GA showed significantly lower IL-1β, IL-6, and TNF-α levels compared with the CSD group. Among the GA-treated groups, reductions were observed consistently in both the serum and hippocampal compartments, with medium- and high-dose GA producing more prominent decreases than the low dose. These findings demonstrate that GA alleviates CSD-induced inflammation across peripheral and central tissues.

### 3.9. Effects of GA on the NF-κB (p65) Signaling Pathway in the Hippocampus of CSD Mice

**Figure 10:**
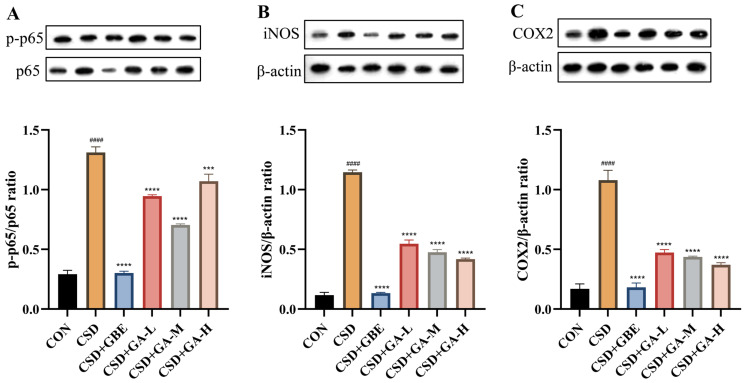
Expression of NF-κB (p65), iNOS, and COX2 in the hippocampus of the CSD mice after GA administration. (**A**) p-p65/p65; (**B**) iNOS/β-actin; (**C**) COX-2/β-actin. Results are shown as the mean ± SEM (*n* = 3). ^####^ *p* < 0.0001 vs. CON; *** *p* < 0.001 and **** *p* < 0.0001 vs. CSD.

To investigate the mechanisms underlying the anti-inflammatory effects of GA, we assessed the NF-κB pathway activation in the hippocampus by examining p-p65, iNOS, and COX2 expression (Figure 10). Compared with the control group, the CSD mice exhibited a markedly increased p-p65/p65 ratio (Figure 10A), as well as elevated levels of iNOS (Figure 10B) and COX2 (Figure 10C), indicating robust activation of the NF-κB signaling pathway under CSD. GBE treatment significantly suppressed these inflammatory markers, restoring them toward control levels. GA supplementation also reduced NF-κB pathway activation: all three doses of GA decreased the p-p65, iNOS, and COX2 expression relative to the CSD group, with more pronounced effects observed in the medium- and high-dose groups. These findings suggest that GA mitigates hippocampal inflammation, partly by suppressing NF-κB signaling pathways.

## 4. Discussion

In this work, we examined the protective role of GA against CSD-induced cognitive impairments and further elucidated its underlying mechanisms of action. Our results revealed three major findings: (1) GA treatment enhanced learning and memory performance in the CSD mice, as evidenced by improvements in the OFT, NOR, ST, and MWM paradigms; (2) GA strengthened antioxidant defenses by elevating T-AOC and SOD activity while reducing MDA levels, along with activation of the Nrf2/HO-1 signaling pathway; and (3) GA mitigated neuroinflammation by lowering circulating IL-1β, IL-6, and TNF-α, as well as by suppressing hippocampal expression of p-p65, iNOS, and COX2. Collectively, these findings provide compelling evidence that GA counteracts CSD-induced cognitive deficits through interconnected antioxidant and anti-inflammatory mechanisms.

CSD is increasingly recognized as a model for simulating common lifestyle-related sleep disturbances, such as shift work, stress, and night-time screen exposure [27]. Compared with acute sleep deprivation paradigms, which capture short-term cognitive changes, CSD more accurately reflects the cumulative and persistent consequences of sleep loss that are relevant to human health [28]. GA administration did not yield a monotonic dose–response in body weight. Instead, a U-shaped pattern emerged, with GA-M values lower than GA-L and GA-H partially returning toward GA-L. Such non-linear variation is consistent with reports that chronic stress models produce heterogeneous growth trajectories due to changes in appetite and metabolism [29] and that polyphenols often display biphasic or U-shaped dose–response relationships [30]. In contrast, GBE maintained body weight close to control levels, suggesting mitigation of stress-induced growth suppression without disrupting normal development. Collectively, these results indicate that body weight should be interpreted as a secondary outcome in this model, whereas the protective effects of GA were more consistently demonstrated in cognitive and biochemical endpoints. The CSD mice exposed to prolonged single-factor stress displayed deficits in cognition-associated behaviors [31].

To evaluate these impairments, we first conducted the NOR test, which capitalizes on the innate drive of rodents to investigate new stimuli, thereby assessing their immediate memory capabilities [32,33]. In line with earlier studies, the CSD mice exhibited a lower discrimination score in the NOR test, thus indicating deficit in recognition memory [34,35]. However, administering GA restored the recognition memory of the mice subjected to CSD. Importantly, total exploration time was also assessed as a parameter commonly used to evaluate locomotor activity and exploratory behavior [36]. Previous studies have reported that chronic stress or sleep deprivation can diminish exploratory drive and reduce total exploration time in the NOR test [37]. GA treatment significantly extended total exploration time, indicating that the improvement in the discrimination index was not due to altered locomotor activity alone but rather reflected a genuine recovery of hippocampal-dependent recognition memory. Next, short-term retention was assessed with the ST test. The CSD mice exhibited shorter latency and more errors, whereas GA administration prolonged latency and reduced errors, demonstrating improved retention. Finally, spatial learning and memory were evaluated with the MWM [38]. Consistent with previous reports, the CSD mice exhibited longer escape latencies during training and fewer platform crossings in the probe trial, which is consistent with the impaired spatial memory described in previous studies [39]. Such findings are in line with reports that chronic sleep deprivation disrupts hippocampal synaptic plasticity and impairs MWM performance, leading to prolonged escape latency and reduced target quadrant preference [40]. GA treatment significantly improved these outcomes, shortening escape latency, increasing target quadrant dwell time, and enhancing platform crossings. These improvements across NOR, ST, and MWM indicate that GA protects hippocampal-dependent learning and memory functions, highlighting its potential for preserving complex cognitive functions under lifestyle-related sleep deprivation.

Oxidative stress occurs when reactive oxygen species (ROS) are generated in excess and redox homeostasis is disrupted [41]. Elevated ROS levels can trigger oxidative damage to lipids, impair protein function, and injure DNA, ultimately leading to synaptic dysfunction and neuronal loss that drive cognitive decline [42,43]. As a pathological process, oxidative stress is strongly associated with neurodegenerative disorders and is particularly evident in CSD [44,45]. Antioxidant enzyme activities such as SOD and T-AOC, along with lipid peroxidation products like MDA, are widely employed as indicators of oxidative stress [46]. Extensive research in both clinical and preclinical settings has repeatedly demonstrated that a lack of sleep significantly increases oxidative stress in the brain—particularly in the hippocampus, where oxidative damage is strongly associated with cognitive deficits [47,48,49]. Consistent with these reports, our study demonstrated that the CSD mice exhibited decreased T-AOC and SOD activity together with elevated MDA levels in both serum and hippocampus, confirming the presence of systemic and central oxidative stress. GA treatment effectively reversed these alterations, restoring antioxidant enzyme activity and reducing lipid peroxidation in both compartments. At the molecular level, GA enhanced Nrf2 activation and its target genes HO-1 and NQO1, counteracting the suppression observed in the CSD mice and reinforcing intrinsic antioxidant defenses [50,51]. Importantly, these findings extend our earlier in vitro results in SH-SY5Y cells to an in vivo context, demonstrating that GA protects against oxidative stress–induced damage under sleep deprivation conditions. Taken together, our study highlights oxidative stress regulation as a core mechanism underlying the cognition-improving effects of GA, and it provides the first evidence that GA simultaneously activates systemic and hippocampal antioxidant defenses in a CSD model, underscoring its translational potential as a dietary polyphenol for neuroprotection.

Neuroinflammation is another key pathological process induced by CSD, often acting synergistically with oxidative stress to exacerbate neuronal dysfunction [52,53,54]. Upon activation of the inflammatory cascade, the p65 subunit of NF-κB translocates into the nucleus, initiating the transcription of major pro-inflammatory mediators such as TNF-α, IL-6, and IL-1β. This process also upregulates the expression of critical downstream effectors, including iNOS and COX-2 [55,56]. Previous studies have reported that chronic sleep deprivation markedly increases peripheral and hippocampal pro-inflammatory cytokines, including IL-1β, IL-6, and TNF-α, thereby linking sleep loss to both systemic and central inflammatory activation [57,58]. Consistent with this, CSD in our study elevated serum and hippocampal IL-1β, IL-6, and TNF-α levels, together with increased hippocampal NF-κB–related proteins, including p-p65, iNOS, and COX-2, thereby confirming both peripheral and central inflammatory activation. GA treatment markedly attenuated these responses by reducing the concentrations of inflammatory cytokines in both serum and in the hippocampus and by suppressing hippocampal NF-κB signaling, indicating that its protective effects involve the inhibition of pro-inflammatory cascades. This is consistent with previous reports showing that GA suppresses cytokine production and NF-κB activation in inflammatory disease models such as ulcerative colitis [59,60], and our findings now extend these effects to a sleep deprivation–related cognitive impairment context. Collectively, these results suggest that GA’s anti-inflammatory activity complements its antioxidant properties, providing dual protection against CSD-induced neuronal injury. The combined modulation of oxidative stress and neuroinflammation underscores GA’s potential as a dietary polyphenol with translational significance for preventing or mitigating the cognitive decline associated with chronic sleep loss.

## 5. Conclusions

Collectively, our data indicate that GA mitigates CSD-induced cognitive deficits through complementary antioxidant and anti-inflammatory actions. Activation of the Nrf2/HO-1 axis restored redox homeostasis, while suppression of NF-κB–mediated cytokine release attenuated neuroinflammatory signaling, yielding integrated neuroprotection against sleep-deprivation–related injury. We acknowledge two limitations: GA concentrations in brain tissue were not measured, and microglial (Iba1/CD68) or astrocytic (GFAP) activation was not directly quantified; future work will establish brain exposure and blood–brain barrier penetration via pharmacokinetic profiling and will apply region-specific hippocampal immunohistochemistry with morphology-based metrics. Within these constraints, this study provides what appears to be the first in vivo evidence that GA protects cognition under CSD, supporting its potential as a dietary polyphenol to mitigate lifestyle-associated cognitive decline.

## Figures and Tables

**Table 1:** Effects of GA on escape latency in the MWM training phase. Data are expressed as the mean ± SEM (*n* = 12). ^##^ *p* < 0.01 and ^####^ *p* < 0.0001 vs. CON; * *p* < 0.05, ** *p* < 0.01, *** *p* < 0.001, and **** *p* < 0.0001 vs. CSD.

Group	D1	D2	D3	D4	D5
CON	60.05 ± 0.02	53.05 ± 0.16	45 ± 0.13	35.65 ± 0.38	22.2 ± 0.54
CSD	60.05 ± 0.02	56.2 ± 1.7	45 ± 0.85	48.8 ± 0.45 ^##^	44.05 ± 0.6 ^####^
CSD + GBE	60.05 ± 0.02	57.05 ± 1.32	36.85 ± 1.32	34.6 ± 0.36 **	24.25 ± 0.29 ****
CSD + GA-L (50 mg/kg)	60.1 ± 0	55.8 ± 1.88	51.2 ± 0.54	38.5 ± 0.89 *	37.4 ± 0.67
CSD + GA-M (100 mg/kg)	60.05 ± 0.02	53.95 ± 2.71	48.45 ± 0.69	40.1 ± 0.18	17.45 ± 0.38 ****
CSD + GA-H (200 mg/kg)	60.05 ± 0.02	56.7 ± 1.48	45.55 ± 0.47	33.75 ± 1.68 ***	20.2 ± 2.41 ****

**Table 2:** Effects of GA on quadrant dwell time in the MWM probe test. Data are expressed as the mean ± SEM (*n* = 12). ^##^ *p* < 0.01 vs. CON; * *p* < 0.05, ** *p* < 0.01, and **** *p* < 0.0001 vs. CSD.

Group	Q1	Q2	Q3	Q4
CON	26.85 ± 0.34	16.3 ± 0.27	16.7 ± 0.4	21.15 ± 0.2
CSD	18.15 ± 0.29	25.7 ± 1.25	34.65 ± 1.81 ^##^	30.1 ± 1.65
CSD + GBE	37.45 ± 3.24 **	18.65 ± 0.87	18.55 ± 0.07 **	23.7 ± 0.45
CSD + GA-L (50 mg/kg)	27.45 ± 1.99	21 ± 0.45	20.25 ± 0.07 *	21 ± 0.27
CSD + GA-M (100 mg/kg)	50.3 ± 3.67 ****	19.8 ± 0.4	27.7 ± 0.76	27.05 ± 0.74
CSD + GA-H (200 mg/kg)	23 ± 1.03	20.15 ± 1.32	15.3 ± 0.27 **	20.6 ± 0.67

## Data Availability

The original contributions presented in this study are included in the article. Further inquiries can be directed to the corresponding authors.

## Abbreviations

The following abbreviations are used in this manuscript:

GA	Gallic Acid
CSD	Chronic Sleep Deprivation
OFT	Open Field Test
NOR	Novel Object Recognition
ST	Step-Through
MWM	Morris Water Maze
T-AOC	Total Antioxidant Capacity
SOD	Superoxide Dismutase
MDA	Malondialdehyde
Nrf2	Nuclear Factor Erythroid 2–Related Factor 2
HO-1	Heme Oxygenase-1
NQO1	NAD(P)H Quinone Dehydrogenase 1
NF-κB	Nuclear Factor Kappa-B
iNOS	Inducible Nitric Oxide Synthase
COX2	Cyclooxygenase-2
IL-1β	Interleukin-1 Beta
IL-6	Interleukin-6
TNF-α	Tumor Necrosis Factor Alpha
